# Share Rose, Get Fun: The Influence of Donation on Happiness

**DOI:** 10.3389/fsoc.2021.675968

**Published:** 2021-12-07

**Authors:** Yuan-yang Wu, Yi-tong Yu, Yi-dan Yao, Mo-han Su, Wen-chao Zhang, Shi-ming Ti, Xue-yu Lin, Shuo Zhang, Si-qing Zhang, Hua-lei Yang

**Affiliations:** School of Public Administration, Zhongnan University of Economics and Law, Wuhan, China

**Keywords:** donation, CGSS, happiness, propensity score matching, OLS

## Abstract

There is little literature on the impact of donation on individual wellbeing in China. This study examines individual donations in China to answer the question of whether helping others makes us happier and to provide policy implications for in Chinese context. Based on the 2012 Chinese General Social Survey (CGSS) data and using ordered logit and OLS as benchmark models, this study finds that donation can significantly increase individual happiness. After using propensity score matching (PSM) to eliminate the possible impact of self-selection, the above conclusion remains robust. After a sub-sample discussion, it is found that this effect is more pronounced under completely voluntary donation behavior, and is not affected by economic factors, indicating that the happiness effect of donation does not vary significantly depending on the individual’s economic status. This study contributes to the literature on donation behavior by examining the impact of donation behavior on donors’ subjective happiness in China, and further identifies subjective happiness differences, as between voluntary and involuntary donations, thereby providing theoretical and empirical support for the formulation of policies for the development of donation institutions in China.

## Introduction

As the cornerstone of China’s philanthropy and one of the most important manifestations of social harmony, the act of donation has gradually become a common phenomenon in the Chinese society. At the same time, the Fourth Plenary Session of the 19th Party Congress also emphasized the importance of donations as a third distribution system and of developing charities and other public welfare undertakings. However, the small amount and low percentage of individual donations is not in line with the state’s active advocacy. A comparison of the donation data between China and the United States over the past 3 years shows that in 2017, total donations in the United States accounted for 2.1% of its GDP and individual donations accounted for 70% of total donations; in the same period, China’s total donations accounted for 0.18% of its GDP and individual donations accounted for 20% of total donations. In 2018, individual donations in the United States accounted for 68% of total donations, and although individual donations in China increased, they only accounted for 23% of the total donations. In 2019, individual donations in China increased 10.54% year-on-year, but corporate donation was still the main source of charitable donation, and in the same period, individual donations in the United States returned to a level of 70% of total donations in 2017. The comparison of individual donation data between China and foreign countries raises questions: Does the act of donation reduce the welfare of residents and hence, people are reluctant to donate? Research on this issue would be beneficial in promoting the development of charitable policies and donation behaviors in China.

Some scholars have investigated donation behavior and individual emotion, social status, and basic characteristic. Based on the influence of individual emotions on donation behavior, Dickert et al. (2011) and Verhaert and Van den Poel (2011) found that there is a correlation between compassion and willingness to donate. Furthermore, Lay et al. (2019) explored the relationship between donation behavior and individual donors’ emotions through a study of 1,300 Chilean participants aged 18–64 years, and found that empathy had a positive influence on donation behavior and personality. Meanwhile, Zhu (2020) found a positive association between donation behavior and life satisfaction. In addition, Nan and Luo (2013) found that the donor’s individual social network and social trust positively contributed to willingness to give, and Winterich et al. (2012) revealed that when the moral foundations of a charity through management processes or mission align with the donor’s political identity, donations increased. In terms of the relationship between individual basic characteristic and individual donation behavior, Noor et al. (2015) concluded from a logistic regression of survey data on Malaysian residents that age, income, education, and religion influence the donation behavior of Malaysian residents, and Su and Shi (2014) found, through a regression analysis of survey data, that residents’ perceptions of charity, altruism, and tax deductions affect their voluntary donations.

In addition, some scholars explored the relationship between individual donation behavior and charitable organizations or programs. Jones et al. (2018), after analyzing a sample of 655 students in an online experimental study, found that negative reports of nonprofit organizations negatively affected donors’ perceptions of such organizations and donation behavior. Liu et al. (2018) argued that the credibility of charitable donation programs was a key factor in donation behavior. Metzger and Günther (2019) also found a positive correlation between the effectiveness of aid information and donation behavior. Wymer and Drollinger (2015) concluded from an analysis of 228 questionnaires collected *via* an online survey panel that the expertise and admiration felt for celebrity spokespeople in charity projects were significant predictors of intention to give.

Pro-social behavior (charitable behavior toward others and the world) also has a positive correlation with happiness, especially that explicit prosocial goals are more likely to increase one’s happiness. Rudd et al. (2014), through a mediation analysis of data collected from respondents on Amazon, argued that the achievement of more specific prosocial goals was more likely to increase people’s happiness. Aknin et al. (2015) found that both adults and children were more likely to feel happy when making donations than when receiving donations in a separate study of children and adults in a small town in Vanuatu. Rowland and Curry (2018) statistically analyzed the survey data of 692 participants, showing that the number of personal charitable activities was positively related to subjective happiness.

After analyzing the international literature, it is evident that although scholars have studied donation behavior, the vast majority of them have studied the factors that influence donation behavior. However, literature on the effects of individual donations on human happiness in China is scarce, and this article attempts to test whether the old Chinese adage that the gift of a rose leaves a legacy is still relevant today. At the same time, without clarifying the impact of donation on the happiness of the population in the Chinese context, we cannot respond to the question of whether the impact of donation on happiness has led to a decrease in individual donation behavior.

The novelty of this study lies in the fact that, unlike studies on the motivations of donation, there are relatively few studies that treat donation behavior as an independent variable to explore its relationship with happiness. This study contributes to the literature on donation behavior by examining for the first time the impact of donation behavior on donors’ subjective happiness in China, and further identifies the differences in subjective happiness between voluntary and involuntary donations, providing theoretical and empirical support for the formulation of policies on donation and industrial development in China. Meanwhile, our study focusing on the relationship between the behavior of donation and individual happiness would inspire scholars in other countries and offer an inspiring look at the behavior of donation among different countries, promoting the research on happiness. The impact of individuals’ participation in donation on their subjective happiness are systematically reviewed and compared with data from outside China, providing a reference for research in the field of donation, and further complementing the existing research on the impact of individual autonomy and economic status on the relationship between donation and happiness.

Based on the above, this study will examine the following questions: First, can individuals’ donation behavior enhance their own happiness? Second, considering the different degrees of autonomy of individuals in making donations, are there any differences in the effects of different autonomous donation behaviors on happiness? Third, considering the important influence of economic factors on individuals’ happiness, is there an effect of differences in economic situation on happiness? To address the above questions, this study explores the relationship between donation and happiness based on the 2012 Chinese General Social Survey data (CGSS 2012), using ordered logit and OLS as benchmark models and conducting a robustness analysis with propensity score matching. On this basis, heterogeneity analysis will be conducted on the autonomy of individuals’ willingness to give and their income status, respectively, to further discuss the influence of autonomy and economic factors on the relationship between donation and happiness.

## Methods

### Data Description

The data used for this study were obtained from the Chinese General Social Survey (CGSS) in 2012 conducted by the National Survey Research Center at Renmin University of China (NSRC) under a network of Chinese academic institutions. Launched in 2003, CGSS was the earliest nationally representative survey to systematically monitor the changing relationship between social structure and quality of life in both urban and rural China. CGSS mainly adopted face-to-face interview when conducting investigation. Chinese General Social Survey (CGSS) selects samples according to certain sampling methods and conducts face-to-face interviews with the selected samples. The respondents are required to truthfully answer questions according to their actual situation. The investigators record the respondents’ answers truthfully and finally integrate the data.

Compared to other years of CGSS, the CGSS in 2012 includes detailed variables on social organization participation, public participation, and social donation, which constitute the basis of the analysis in this paper. CGSS2012 uses a multi-order stratified probability sampling design, and the survey covers all provincial administrative units in China (excluding Hong Kong, Macau, and Taiwan). A total of 480 committees (district and village) were surveyed across the country, with 25 households surveyed in each committee. In this study, 5,819 data points were obtained after variable filtering and data cleaning. The distribution of data is shown in Table 1 below. In the whole sample, about 31.9% of individuals engaged in donation activities. The gender distribution of men and women was relatively balanced. The average age of the sample was 48 years old, and was in middle age. The self-reported health status of most respondents was relatively good. About 79.3% of the individuals had spouses, most of them had one to two children, a large number of individuals participated in pension and medical insurance, and most of them live in rural communities.

**TABLE 1:** Summary statistics.

Variable	Total					Donors		Non-donors	
	Sample	Mean	Standard deviation	Min	Max	Mean	Standard deviation	Mean	Standard deviation
Happiness	5,797	3.824	0.845	1	5	3.974***	0.799	3.753	0.856
Donation	5,819	0.319	0.466	0	1				
Gender	5,819	0.502	0.500	0	1	0.488	0.500	0.509	0.500
Age	5,818	48.92	16.43	17	94	44.976***	15.5	50.766	16.53
Age square	5,818	2,663.04	1,667.982	289	8,836	2,262.975***	1,496.177	2,850.302	1710.917
Minority	5,815	0.087	0.281	0	1	0.098**	0.297	0.082	0.274
Self-reported health	5,818	3.535	1.079	1	5	3.710***	1.006	3.454	1.102
Marriage	5,819	0.793	0.405	0	1	0.809**	0.393	0.785	0.411
Child	5,814	1.813	1.379	0	10	1.474***	1.130	1.972	1.454
Religion	5,818	0.144	0.351	0	1	0.164***	0.371	0.134	0.341
Education	5,816	8.718	4.669	0	19	10.636***	4.209	7.821	4.603
Ln(income)	5,266	8.61	2.882	0	13.122	9.126***	2.809	8.357	2.884
In party	5,808	0.120	0.325	0	1	0.183***	0.387	0.090	0.286
In work	5,819	0.636	0.481	0	1	0.671***	0.470	0.62	0.485
Medical	5,778	0.909	0.288	0	1	0.930***	0.256	0.899	0.301
Pension	5,715	0.662	0.473	0	1	0.701***	0.458	0.645	0.479
In urban	5,812	0.234	0.424	0	1	0.410***	0.492	0.152	0.359

∗ *p* < 0.1. ∗∗ *p* < 0.05. ∗∗∗ *p* < 0.01.

#### Happiness

This refers specifically to subjective happiness and people’s evaluation of the happiness in their own lives, including emotional and cognitive aspects (Diener, 2000). In addition, referring to Wang H. (2015), a direct holistic assessment of happiness was used to measure subjective happiness. Here, the variable was measured by asking a question, “How happy do you feel about your life as a whole these days?” The answers were scored from 1 to 5 (1 = *very unhappy*, 2 = *unhappy*, 3 = *neither happy nor unhappy*, 4 = *happy*, 5 = *very happy*). This question is a comprehensive measure of respondents’ life happiness, including both long-term life wellbeing and the impact of recent life events.

#### Acts of Donation

This variable was measured by asking the question, “In 2011, have you personally made a donation to society in the form of money, in kind, or ownership? In this context, we refer to donations that you have made to individuals or organizations in the community voluntarily and without the intention of receiving a donation back.” We set the value of this variable to 1 for participating donations and to 0 for non-participating donations. In fact, the questionnaire asked the respondents about their behavior of donation in 2011. However, there would be no bias caused by large time difference, because the variable of happiness was generated for wellbeing in 2011.

Taking into account the influence of other factors on happiness as per Wang J. D. (2015), this study selected the interviewees’ demographic characteristics (gender, age, marriage, education, and self-reported health), socioeconomic characteristics (personal annual income, political affiliation, work status, medical insurance, and pension insurance), and social contact characteristics (religion and place of residence) as control variables. Based on Liu (2019), integration of ethnic minorities into society has a positive effect on happiness, so demographic characteristics ethnicity is also included in the model as a control variable. Meanwhile, following the suggestion by Rosenbaum and Rubin (1983), a higher-order term of $\;x_{i}\;$ (the square of age) is introduced into the model to improve the flexibility of the equation format, and hence the accuracy of the calculation results. The details are listed in Table 2.

**TABLE 2:** Description of control variables.

Variable name	Meaning
Happiness	very unhappy = 1, unhappy = 2, neither happy nor unhappy = 3, happy = 4, very happy = 5
Donation	donor = 1, non-donor = 0
Gender	male = 1, female = 0
Age and its square	age of respondent in 2012
Minority	ethnic minority = 1, Han Chinese = 0
Self-reported health	very unhealthy = 1, unhealthy = 2, general = 3, healthy = 4, very healthy = 5
Marriage	cohabitation, first marriage with a spouse, remarriage with a spouse = 1, unmarried, separation without divorce, divorced, widowed = 0
Child	The number of children the respondent has
Religion	whether the respondent has religious belief; if yes, religion = 1, otherwise religion = 0
Education	no education = 0, kindergarten = 3, primary school = 6, junior high school = 9, secondary vocational technical school or technical school = 11, high school or technical secondary school = 12, junior college (adult, formal) = 15, undergraduate (adult, formal) = 16, Graduate student or above = 19
Ln(income)	natural logarithm of respondents’ total income in 2011
In party	member of the Chinese Communist Party or Democratic Party = 1; non-party members = 0
In work	employed = 1, unemployed = 0
Medical	insured= 1, uninsured = 0
Pension	insured = 1, uninsured = 0
In urban	resident committee = 1, village committee = 0

### Empirical Strategy

Whether an individual participates in public welfare behaviors such as donations is highly influenced by his individual characteristics, as it is a self-selective behavior. For example, people from good families and with higher subjective happiness are more likely to participate in philanthropic activities, such as donations. Such people made up the majority of the sample, leading to the high levels of happiness that we measured. If the choice to participate in donations was randomly determined, there would be no selection dilemma. However, since the initial conditions of donors and non-donors are different, the effect of donation behavior on happiness in the basic regression results may not have internal validity if this issue is not addressed effectively. To effectively mitigate self-selection bias, this study used propensity score matching for treatment.

The specific steps of propensity score matching are as follows: Based on the key explanatory variable $\;donate_{i}$ , the donors in the sample were classified into the treatment group and the non-donors were classified into the control group. We then found as many control variables as possible that affected both the dependent variable $happiness_{i}\;$ and the key explanatory variable $\;donate_{i}$ . We selected the demographic characteristics, socio-economic characteristics, and social contact characteristics as control variables. Next, we used probit or logit models to estimate the propensity scores. Finally, based on the propensity score, we used three different matching methods for matching: *K*-nearest neighbor match (*K* = 5), radius match (radius = 0.01), and kernel match. The results were used to compare the differences between the treatment and control groups after matching and obtaining ATT. The formula follows.

$$\text{ATT}=\text{E}\left[{\text{happiness}}_{1\text{i}}{\text{ | D}}_{\text{i}}=1,\text{p}\left({\text{X}}_{\text{I}}\right)\right]-\text{E}\left[{\text{happiness}}_{0\text{i}}{|\text{D}}_{\text{i}}=0,\text{p}\left({\text{X}}_{\text{i}}\right)\right],$$ (1)

Notes: $happiness_{1i}\;$ is the happiness of the treatment group and $happiness_{0i\;}$ is the happiness of the control group. $\;D_{i}$ is the explanatory variable, which refers to $donate_{i}$ , such that $\;D_{i}=1\;$ means the individual is a donor and $D_{i}=0$ means the individual is a non-donor, and $p\left(X_{i}\right)$ is the propensity score, representing the probability that the individual participates in donation. This study uses the logit model to estimate propensity scores.

**FIGURE 1:**
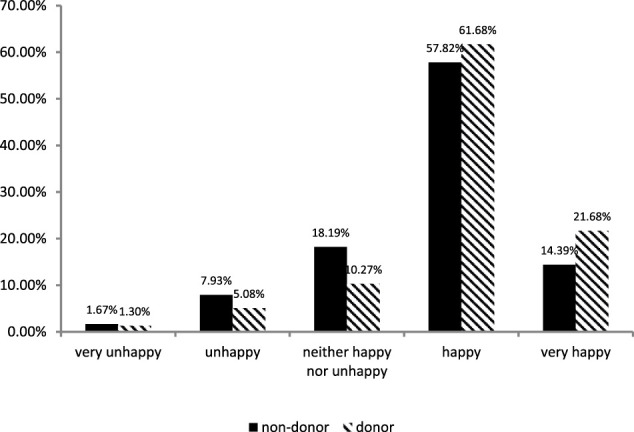
Distribution of happiness based on CGSS 2012 data.

From Figure 1, it can be seen that the distribution of people who are *very unhappy*, *unhappy*, and *neither happy nor unhappy* is higher among non-donors than among donors, while among the *happy* and *very happy* groups, the distribution of donors was higher than the non-donors. Combined with the summary statistics, we can see that the donors have a higher sense of happiness, and we can infer that there may be a relationship between donation and happiness.

## Results and Discussion

### Basic Regressions on Outcomes

First, this study estimates the basic equations of happiness determination using ordered ordinal logistic regression and OLS regression. The regression outcomes of the regressions are presented in Table 3. As seen from the outcomes in the table, for the key explanatory variable donation behavior, the results of the ordered logit and OLS regressions are consistent in terms of direction and significance. This indicates that the act of donation can increase people’s happiness, and this effect is positive and significant at the 1% level. Meanwhile, the higher the total value of donation, the higher the individuals’ happiness. There was a positive relationship between the value of donation and happiness.

**TABLE 3:** Basic regressions on outcomes.

	(1)	(2)	(3)	(4)
	Happiness	Happiness	Happiness	Happiness
Donation	0.520***	0.455***	0.218***	0.175***
	(0.094)	(0.104)	(0.037)	(0.039)
Total value of donation	0.0000552**	0.0000413**	0.0000218***	0.0000157***
	(0.000)	(0.000)	(0.000)	(0.000)
Male		−0.156***		−0.061**
		(0.059)		(0.024)
Age		−0.062***		−0.026***
		(0.012)		(0.005)
Age square		0.001***		0.000***
		(0.000)		(0.000)
Minority		−0.057		−0.039
		(0.111)		(0.044)
Self-reported health		0.475***		0.189***
		(0.033)		(0.013)
Marriage		0.503***		0.210***
		(0.082)		(0.035)
Child		0.046		0.016
		(0.031)		(0.013)
Religion		0.146		0.023
		(0.096)		(0.039)
Education		0.017*		0.010***
		(0.009)		(0.004)
Ln(income)		0.036***		0.016***
		(0.012)		(0.005)
In party		0.209**		0.075**
		(0.085)		(0.033)
In work		−0.283***		−0.092***
		(0.079)		(0.032)
Medical		0.269**		0.121***
		(0.107)		(0.046)
Pension		0.037		0.022
		(0.067)		(0.028)
In urban		−0.183***		−0.071***
		(0.067)		(0.027)
Constant term			3.803***	3.240***
			(0.012)	(0.134)
Intercept term1	−4.111***	−2.961***		
	(0.106)	(0.349)		
Intercept term2	−2.327***	−1.108***		
	(0.047)	(0.333)		
Intercept term3	−1.097***	0.129		
	(0.031)	(0.331)		
Intercept term	1.666***	3.084***		
	(0.037)	(0.336)		
*N*	5,797	5,142	5,797	5,142
*R* ^2^			0.007	0.087

①Standard deviations are shown in parentheses.②. ∗ *p* < 0.1. ∗∗ *p* < 0.05. ∗∗∗ *p* < 0.01.

For the control variables, in terms of individual characteristics, gender was significant, and men were less happy than women. Age is significant, and the squared term of age is also significant, implying a “U-shaped relationship” between age and happiness. In terms of health status, the higher the level of health, the higher the sense of happiness. Among family factors, marriage has a clear positive effect on happiness. In terms of socioeconomic factors, individuals with better income status have higher happiness. Individuals insured by health insurance also have increased happiness. In the case of work status, there was a significant decrease in happiness for individuals who had a job compared to individuals who did not have a job. In terms of social participation, the happiness of individuals who participated in social organizations or groups increased significantly. In terms of the living environment, the happiness level of rural residents was higher.

### Propensity Score Matching Regression Analysis

#### Propensity Score Matching Method Applicability Test

As the issue of self-selection is not considered in the ordered logit and OLS models, selection bias may exist. This study applies propensity score matching for robustness testing to control for differences in the characteristics of individual donors and non-donors. According to Eq. 1, based on the interviewees’ demographic characteristics, socio-economic characteristics, and social contact characteristics, we calculated the propensity scores. To ensure the matching results, a balance test was conducted in this study, and the density function distributions before and after matching were also reported.

The results of the balance tests are presented in Table 4. The standardized bias of all variables except gender was significantly lower after matching, and even though the deviation of the gender variable increased slightly after matching, overall, the standardized deviation of all variables was controlled within the desired 10% after matching. The difference between the treatment and control groups was not significant, indicating a better matching effect.

**TABLE 4:** Balance test.

Variable	Mean				Deviation reduction ratio (%)	*t*-test	
	Sample	Treatment group	Control group	Deviation rate (%)		*t*-value	p>|t|
Male	U	0.501	0.516	−3.1	−9.8	−1.06	0.291
	M	0.502	0.485	3.4		1.00	0.318
Age	U	45.612	51.234	−35.9	96.4	−11.98	0.000
	M	45.702	45.498	1.3		0.39	0.696
Age square	U	2,313	2,883.4	−35.9	96.2	−11.86	0.000
	M	2,321.1	2,299.3	1.4		0.43	0.668
Minority	U	0.099	0.084	5.2	84.1	1.76	0.000
	M	0.096	0.099	−0.8		−0.23	0.815
Self-reported health	U	3.689	3.435	23.9	96.7	7.95	0.000
	M	3.684	3.692	−0.8		−0.23	0.815
Marriage	U	0.828	0.799	7.4	75.4	2.48	0.013
	M	0.828	0.821	1.8		0.54	0.587
Child	U	1.508	1.975	−36.5	99.9	−11.81	0.000
	M	1.511	1.510	0.0		0.01	0.988
Religion	U	0.161	0.129	8.9	60.1	3.04	0.002
	M	0.157	0.145	3.5		1.01	0.313
Education	U	10.6	7.797	64.3	100.0	21.40	0.000
	M	10.565	10.565	0.0		−0.00	1.000
Ln(income)	U	9.129	8.368	26.8	80.2	8.98	0.000
	M	9.121	8.971	5.3		1.58	0.114
In party	U	0.192	0.096	27.7	95.7	9.84	0.000
	M	0.188	0.192	−1.2		−0.31	0.759
In work	U	0.691	0.635	11.7	63.5	3.92	0.000
	M	0.689	0.669	4.3		1.26	0.210
Medical	U	0.933	0.906	9.7	100.0	3.18	0.002
	M	0.932	0.932	0.0		−0.00	1.000
Pension	U	0.713	0.656	12.1	97.9	4.05	0.000
	M	0.711	0.710	0.3		0.08	0.939
In urban	U	0.728	0.545	38.8	99.4	12.84	0.000
	M	0.727	0.726	0.3		0.08	0.938

The table reports the *p-*value of the *t*-test for equality of means in the treated and control groups and the percentage of bias and its reduction that is the standardized bias. For each variable, the indicators are calculated before and after matching. “M” means the sample was matched, “U” means the sample was not matched.

**FIGURE 2:**
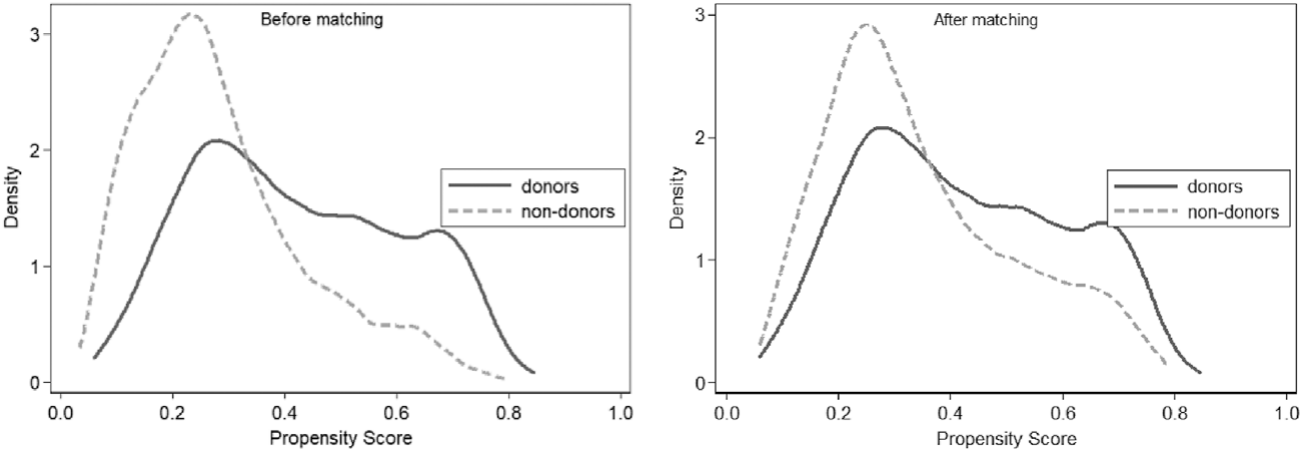
Density function plots.

Figure 2 shows the density function plots of the treatment group and the control group before (left) and after matching (right). It can be clearly seen that the two lines of the treatment group and the control group after matching overlap more and differ less than before matching, which can also indicate that the matching is more effective. The results of the balance test also indicate that the propensity score matching method is more suitable.

#### Propensity Score Matching Regressions on Outcomes

After eliminating the self-selection of donation behavior, this study attempts to select the most appropriate matching method to examine the treatment effect of donation behavior on wellbeing. Although there is no consensus among academics on which matching method will yield optimal results, according to Chen (2014), if the results obtained after applying multiple matching methods are similar or even consistent, it means that the matching results are robust and the sample validity is good. Therefore, this study selects three mainstream methods for matching: *K*-nearest neighbor match (*k* = 5), radius match (with caliper set to 0.01), and kernel match.

The results are presented in Table 5, showing that the ATT of donors compared to non-donors in the three matching methods increased by 0.126, 0.099, and 0.124, respectively, and all of them were significant at the 1% level, which was slightly lower than the baseline regression results, but it is undeniable that donation behavior has a positive effect on wellbeing. It can be seen that even though the ATT of radius matching is slightly smaller than for the other two matching methods, the overall results of the model are basically the same, indicating that the results of this study are robust. Comparing the ATTs before and after matching shows that the results all decrease significantly after matching, indicating that the positive effect of participation in donation behavior on happiness is overestimated without taking self-selection into account.

**TABLE 5:** Propensity score matching regressions on outcomes.

Variable	Sample	Donors	Non-donors	ATT	Standard deviation
*K*-nearest neighbor match (*K* = 5)	U	3.981	3.772	0.209***	0.025
	M	3.977	3.877	0.126***	0.035
Radius match	U	3.981	3.772	0.208***	0.025
	M	3.977	3.877	0.099**	0.039
Kernel match	U	3.981	3.772	0.208***	0.025
	M	3.977	3.853	0.124***	0.025

Note: ① Robust standard errors are in parentheses.②. ∗ *p* < 0.1. ∗∗ *p* < 0.05. ∗∗∗ *p* < 0.01.③. “M” means the sample was matched, “U” means the sample was not matched.

For the overall matching effect, the impact of donation behavior on happiness was positive and significant, which is consistent with earlier results. Mu (2005) points out that for an act to be voluntary in nature, it must have at least three characteristics: voluntariness, gratuitousness, and public welfare. According to the definition of donation in the questionnaire, it is clear that the donation behavior in this study meets the basic characteristics of voluntary behavior. Lee (2019) pointed out that the positive connection between voluntary behavior and happiness can be explained at psychological and social levels, whereby individuals can form a sense of being valued, have their self-esteem satisfied, become more aware of the value of life, and gain a sense of wellbeing through voluntary donation. At the social level, based on Xing and Zhang (2019), all five dimensions of social cohesion have a positive effect on residents’ happiness. Among them, the effects of social trust and social belonging were more obvious. Donation of a voluntary nature can promote the integration of individuals into society, enhance the bond between individuals and society through social interaction with others, and gain social support, thereby creating a sense of social trust and social belonging for individuals and in turn enhancing their own sense of wellbeing. The World Giving Index (WGI), measured and published annually by the Charities Aid Foundation since 2010, includes “donation behavior” as one of the indicators of pro-social behavior. As a performer of this pro-social behavior, donors can obtain the satisfaction of three basic needs (Gagné 2003): the need for a sense of competence, which inspires a stronger sense of efficacy in the donor; the need for relationalism, which makes the donor feel valued; and the need for autonomy, which allows the donor to experience firsthand the sense of self-control that comes with helping others. Wang J. D. (2015) pointed out that contemporary people need to pay more attention to the relationship between consumption and happiness, and increasing spiritual consumption represented by donation can bring people psychological and spiritual abundance and fundamentally improve residents’ happiness. Overall, the effect of individual giving as an important form of volunteering to enhance subjective wellbeing is consistent in Chinese and foreign studies.

## Further Discussion: Heterogeneity Analysis

### Initiative of Donation

Although individuals do not typically make voluntary donations for the purpose of return, the motivation of individual donation can vary, such that the autonomy of individual participation in the behavior differs as a voluntary service and is not entirely initiated by individuals, but relies on the promotion of administrative forces. According to the Q part of CGSS 2012, “Among all the donations you made in 2011, according to the degree of autonomy, do you have the following three types of donations?” Among them, this paper defines “voluntary donation initiated by the government or units” as “semi-voluntary” and “mandatory donation by the government and units” as “mandatory.” Finally, the samples were divided into three categories: “fully voluntary,” “semi-voluntary,” and “mandatory.” In this paper, “fully voluntary” is defined as voluntary donation, while “semi -voluntary” and “mandatory” are defined as externally driven donations. It was worth noting that respondents are required to give only one answer to the question. So, respondents can only choose one of voluntary and compulsory donations to answer. There were no overlapping answers.

There are significant differences in the results for fully voluntary donation behavior and semi-voluntary and mandatory participation in donation. There are positive effects on happiness in the full voluntary group, but the positive effects in semi-voluntary and mandatory group are not significant at the 10% statistical level. Comparing the coefficients of the three types of groups, it is clear that fully voluntary donation has a more significant effect on happiness than the other two types in Table 6. This can be interpreted to mean that the less restricted and more spontaneous one’s own donation behavior, the more significant the increase in happiness (Table 7).

**TABLE 6:** Discussion of different degree of Autonomy.

	Order logit			OLS		
	(5)	(6)	(7)	(8)	(9)	(10)
	Fully voluntary	Semi voluntary	Mandatory	Fully voluntary	Semi voluntary	Mandatory
Donation	0.454^***^	0.036	0.185	0.174^***^	−0.011	0.065
	(0.069)	(0.088)	(0.203)	(0.027)	(0.035)	(0.078)
Male	−0.071	−0.081	0.141	−0.026	−0.015	0.032
	(0.125)	(0.167)	(0.418)	(0.047)	(0.062)	(0.141)
Age	−0.086***	−0.052	−0.219**	−0.032***	−0.025	−0.100***
	(0.028)	(0.041)	(0.107)	(0.010)	(0.016)	(0.036)
Age square	0.001***	0.001*	0.003**	0.000***	0.000**	0.001***
	(0.000)	(0.000)	(0.001)	(0.000)	(0.000)	(0.000)
Minority	0.222	−0.219	0.394	0.079	−0.118	0.055
	(0.219)	(0.336)	(0.702)	(0.078)	(0.135)	(0.194)
Self-reported health	0.512***	0.542***	0.446*	0.191***	0.178***	0.145
	(0.076)	(0.102)	(0.252)	(0.027)	(0.040)	(0.091)
Marriage	0.382**	0.110	−0.317	0.169**	0.021	0.114
	(0.188)	(0.274)	(0.857)	(0.073)	(0.110)	(0.290)
Child	0.031	−0.002	−0.638**	0.013	−0.014	−0.205*
	(0.075)	(0.108)	(0.282)	(0.027)	(0.043)	(0.109)
Religion	0.181	0.485	0.635	0.063	0.087	0.150
	(0.175)	(0.313)	(0.566)	(0.063)	(0.122)	(0.220)
Education	0.003	0.036	0.030	0.006	0.014	0.018
	(0.019)	(0.029)	(0.072)	(0.007)	(0.011)	(0.026)
Ln(income)	0.037	0.075	0.013	0.012	0.032*	0.000
	(0.025)	(0.048)	(0.104)	(0.009)	(0.018)	(0.033)
In party	0.152	0.103	0.757**	0.063	0.013	0.288**
	(0.166)	(0.196)	(0.376)	(0.063)	(0.073)	(0.124)
In work	−0.174	−0.142	−0.243	−0.039	−0.008	0.046
	(0.186)	(0.294)	(0.601)	(0.071)	(0.110)	(0.237)
Medical	0.230	0.513	−0.243	0.096	0.206	0.100
	(0.246)	(0.396)	(1.044)	(0.098)	(0.172)	(0.377)
Pension	0.033	−0.444*	−0.735	0.036	−0.111	−0.186
	(0.150)	(0.227)	(0.488)	(0.058)	(0.088)	(0.152)
In urban	−0.109	0.150	−0.315	−0.047	0.045	−0.185
	(0.149)	(0.237)	(0.461)	(0.055)	(0.089)	(0.180)
Constant term				3.518***	3.050***	5.491***
				(0.266)	(0.399)	(0.658)
Intercept term1	−3.643***	−1.907*	−8.417***			
	(0.789)	(1.013)	(2.124)			
Intercept term2	−1.938***	−0.025	−7.234***			
	(0.746)	(1.017)	(2.132)			
Intercept term3	−0.757	1.041	−6.135***			
	(0.734)	(1.010)	(2.105)			
Intercept term4	2.307***	4.237***	−2.686			
	(0.740)	(1.030)	(2.078)			
*N*	1,171	655	144	1,171	655	144
*R* ^2^				0.076	0.084	0.168

Note: ① Robust standard errors are in parentheses. ② ∗ *p* < 0.1. ∗∗ *p* < 0.05. ∗∗∗ *p* < 0.01.

**TABLE 7:** Discussion of different income level.

	Order logit		OLS	
	(11)	(12)	(13)	(14)
	Good income	General income	Good income	General income
Donation	0.469***	0.406***	0.142***	0.157***
	(0.084)	(0.100)	(0.030)	(0.043)
Male	−0.119	−0.261***	−0.034	−0.114***
	(0.089)	(0.085)	(0.032)	(0.038)
Age	−0.067***	−0.050***	−0.025***	−0.023***
	(0.018)	(0.016)	(0.006)	(0.007)
Age square	0.001***	0.001***	0.000***	0.000***
	(0.000)	(0.000)	(0.000)	(0.000)
Minority	0.484***	−0.276**	0.178***	−0.131**
	(0.182)	(0.129)	(0.060)	(0.058)
Self-reported health	0.480***	0.450***	0.157***	0.203***
	(0.051)	(0.044)	(0.018)	(0.018)
Marriage	0.597***	0.394***	0.223***	0.183***
	(0.130)	(0.104)	(0.049)	(0.049)
Child	0.088	0.017	0.023	0.008
	(0.056)	(0.036)	(0.021)	(0.017)
Religion	0.392***	−0.101	0.113**	−0.075
	(0.141)	(0.127)	(0.049)	(0.057)
Education	−0.007	0.022*	0.001	0.011*
	(0.014)	(0.013)	(0.005)	(0.006)
Ln(income)	0.320***	0.024*	0.121***	0.010*
	(0.081)	(0.013)	(0.028)	(0.006)
In party	0.252**	0.087	0.079**	0.049
	(0.105)	(0.158)	(0.037)	(0.068)
In work	−0.353***	−0.240**	−0.118**	−0.076*
	(0.136)	(0.100)	(0.049)	(0.045)
Medical	0.287*	0.199	0.133**	0.086
	(0.166)	(0.135)	(0.066)	(0.063)
Pension	−0.008	0.074	0.015	0.031
	(0.110)	(0.083)	(0.039)	(0.038)
In urban	−0.327***	−0.168**	−0.120***	−0.076**
	(0.111)	(0.086)	(0.040)	(0.038)
Constant term			2.344***	3.157***
			(0.321)	(0.194)
Intercept term1	−1.015	−2.580***		
	(0.951)	(0.457)		
Intercept term2	1.209	−0.837*		
	(0.927)	(0.444)		
Intercept term3	2.727***	0.252		
	(0.921)	(0.442)		
Intercept term4	5.882***	3.079***		
	(0.928)	(0.449)		
*N*	2,574	2,568	2,574	2,568
*R* ^2^			0.088	0.087

Note: ① Standard deviations in parentheses. ② ∗ *p* < 0.1. ∗∗ *p* < 0.05. ∗∗∗ *p* < 0.01.

In light of China’s national conditions, the following aspects should be taken into account to improve the autonomy of individuals in donation: On the one hand, when advocating donation activities, we should minimize the occurrence of compulsory donation activities, such as collective donation activities organized by the company, and more donation activities should be held in the form of individual voluntary donation. On the other hand, donation behavior has a positive effect on individual wellbeing. We should improve the individual’s happiness perception in the donation process and give appropriate spiritual encouragement and honor recognition to the individuals engaged in donation, so as to improve the happiness perception.

### Differences in Income Status

Next, heterogeneity was analyzed by income status, and the participants were divided into two groups, with individuals above the median in the “good income status” group and those below the median in the “average income status” group. Here, again, ordered logit and OLS were used to test for different income statuses separately, and the results are shown in Table 7.

The behavior of donation has a positive effect on happiness whether in a good income group or in a general income group. That is to say, when we explore the relationship between the donation and happiness, the happiness generated by donation is more obvious than the happiness brought by high level of income. As we know, when we only analyze the relationship between income and happiness, the impact of income on happiness was hard to ignore. However, according to the results in Table 7, the impact of donation on individuals’ happiness was not influenced by the different level of income.

## Conclusion

In addition to government and market forces, individual and institutional charitable donations have become an indispensable force for social welfare, and the social role of donation behavior has received widespread attention. In this study, based on the 2012 Chinese General Social Survey (CGSS 2012), we explore whether the donation behavior of Chinese individuals contributes to their level of happiness. In this study, we use ordered logit and OLS as the baseline model, and propensity score matching to eliminate the effect of self-selection in order to further measure the effect of donation behavior on happiness.

The results show the following: (1) Both the ordered logit and OLS models suggest that the donation behavior of Chinese individuals contributes to their happiness level, and the findings still hold true after eliminating the effect of self-selection using propensity score matching. (2) Since there may be differences in the autonomy of individuals to make donations, this study uses the questionnaire on autonomy to classify the sample behaviors into “fully voluntary,” “semi-voluntary,” and “mandatory.” The results show that fully voluntary donation behaviors are not as autonomous as those that are not but are associated with a significantly higher increase in happiness level than the other two categories. (3) In this study, the median income of individuals was used as the boundary, and the sample was divided into the two categories of good and average income to explore the impact of donation behavior on the happiness of individuals with different income statuses. In the regression results, the impact of donation on individuals’ happiness was not influenced by the different level of income.

The significance of this study is that it adds to the discussion of the autonomy of individual donation behavior and the difference in the effect of donation on happiness at different income levels, based on a study of the effect of individual donation behavior on happiness levels. The policy implication of this study is that the moral education sector should strengthen the cultivation of autonomous motivation of the perpetrators of pro-social behaviors such as donation, enhance social cohesion, and cultivate a favorable atmosphere for charitable donations in society, and change the role of the government or institutions from the promoter of social donation to the escort of individual donation, so that people’s donation behavior is not passive. As the saying goes, “A rose is a gift that leaves a fragrance in the hand.” This study finds that the happiness of donors comes mainly from their autonomous motivation, and that fully autonomous donation behavior significantly enhances happiness, while the impact of income status becomes secondary. In the WGI ranking mentioned earlier, China does not score well, which seems to run counter to the traditional Chinese virtue of “helping others.” This study demonstrates that “helping others” is not bad in the Chinese context, but that it is important to increase the motivation to help others, to foster public volunteerism, and to increase the role of the government and relevant institutions in escorting social donation. The policy recommendations in this paper are to create a favorable atmosphere for volunteering to help people in society, such as setting up charity activity weeks in the community and promoting the spirit of charity with the help of Lei Feng Day and others. To improve relevant laws and regulations related to charity volunteering and provide legal protection for public participation, the government needs to introduce matching measures to form a multi-level, multi-disciplinary, systematic, complete, and professional legal system for volunteering (Dang 2019). The government should “de-bureaucratize” fundraising activities, ensure the openness and transparency of fundraising information and the use of fundraising funds, and meet people’s needs for diversified donations so that individual donations do not disappear. Individual donations have made a significant contribution to the war against the pandemic in China, inspiring the nation to fight the pandemic with determination and becoming an important supporting force in this major public health emergency.

## Limitations

Although this paper demonstrated the relationship between donation behavior and wellbeing in detail, there are still some limitations that can be further improved. First, the shortcoming of this study was that it did not go further to conduct an in-depth quantitative analysis of the cost of donation and discuss the impact of the cost of individual donation on happiness. Second, we did not consider the relationship between the frequency of donations and wellbeing. At present, the data of donation frequency were still very limited, so it was difficult to carry out empirical research. As we know, different forms of donation such as money and ownership may have different impact on happiness. The different forms of donation was also not considered in our models due to the lack of data. Third, a happy event or a sad event, such as the loss of a friend or relative, had a significant impact on the participants’ happiness. When we evaluated the impact of donation on happiness, we cannot eliminate the impact of these events on individuals’ happiness.

## Data Availability

The datasets for this study can be found in the Chinese General Social Survey(CGSS) (http://cgss.ruc.edu.cn/). Please see http://cgss.ruc.edu.cn/ for more details.
